# Supplementary material for: PSME4 Degrades Acetylated YAP1 in the Nucleus of Mesenchymal Stem Cells
Source: Pharmaceutics. 2022 Aug 9;14(8):1659. doi: 10.3390/pharmaceutics14081659 (PMC9415559; doi:10.3390/pharmaceutics14081659)
Supplement: Supplementary file 1 [file pharmaceutics-14-01659-s001.zip › pharmaceutics-1796135-supplementary.pdf]

SUPPLEMENTARY FIGURES AND FIGURE LEGENDS

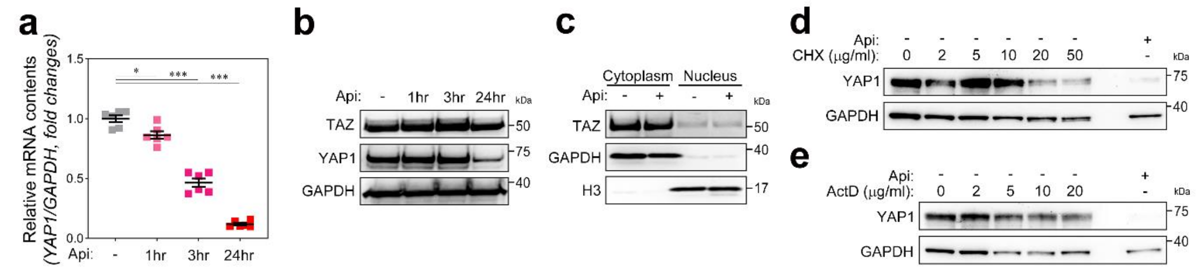

Supplementary Figure S1. Apicidin induces transcription arrest and protein degradation simultaneously. (a) Apicidin started to arrest transcription of YAP1 as early as 3 hours after treatment and totally blocked transcription after overnight treatment in hTERT-MSCs. (b) Apicidin failed to regulate TAZ in cardiac commitment. TAZ remained unchanged while YAP1 was almost removed by apicidin treatment. (c) TAZ did not undergo subcellular redistribution even in the presence of apicidin. The cytoplasmic fraction was obtained by use of hypotonic buffer and the nuclear fraction by use of hypertonic buffer. GAPDH was utilized as a marker for cytoplasm and Histone H3 as a loading control for nuclear protein. (d and e) Acute loss of YAP1 mediated by apicidin treatment was much faster than natural turnover. Overnight inhibition of *de novo* synthesis either by cycloheximide (translation inhibitor, D) or actinomycin D (transcription inhibitor, E) was weaker than apicidin-induced YAP1 ablation, which implied that apicidin promoted active clearance beyond simple transcriptional arrest. Data are presented as mean ± standard error. One-way analysis of variance (ANOVA) with Tukey's Honestly Significant Difference (HSD) post hoc test (A). Asterisks: * $p<0.05$. *** $p<0.001$. Abbreviations: ActD, actinomycin D; Api, apicidin; CHX, cycloheximide; hr, hours.

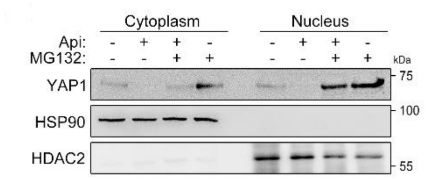

Supplementary Figure S2. Fractional western blot. The subcellular fraction was obtained by use of hypotonic buffer (for cytoplasm) and hypertonic buffer (for nucleus). Proteasomal inhibitor, MG132, predominantly preserved apicidin-induced YAP1 degradation in the nucleus. HSP90 shows the cytoplasmic fraction and HDAC2 the nuclear fraction.

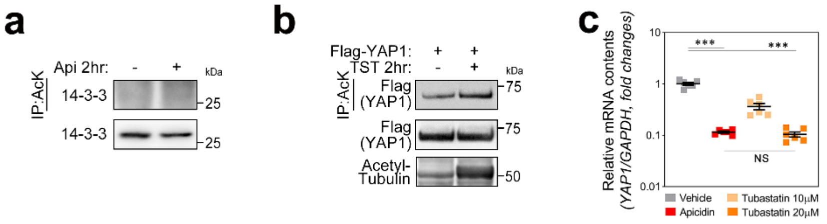

Supplementary Figure S3. Tubastatin A, a selective HDAC6 inhibitor, phenocopies induced by apicidin. (a) Apicidin did not induce acetylation of 14-3-3. (b) Tubastatin A (TST) successfully acetylated YAP1. Acetylation of YAP1 was significantly induced by 2 hours of treatment with TST. Acetyl-tubulin represents inhibition of cytosolic HDACs. (c) Overnight treatment of TST arrested YAP1 transcription as effective as apicidin. Data are shown as mean ± standard error. One-way ANOVA with Tukey's HSD multiple comparison (c). Asterisks *** $p<0.001$. Abbreviations: AcK, acetyl-lysine; hr, hours.

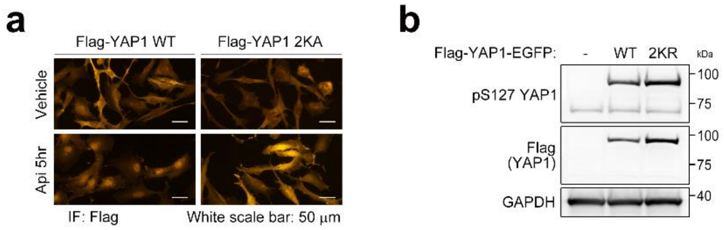

Supplementary Figure S4. Acetylation determines YAP1 localization in a phosphorylation-independent manner. (a) Immunofluorescence for YAP1. Wild-type or acetylation-resistant mutant of YAP1 was visualized with immunofluorescence by Flag antibody. Five hours of treatment was enough to localize YAP1 wild-type into the nucleus, but the acetylation-dead mutant of YAP1 remained in the cytoplasm. (b) Phosphorylation S127 of YAP1 was not altered by acetylation status. Note that no difference was observed between the wild-type and acetylation-resistant mutant. Abbreviations: 2KA, substitution of lysines (K) 494/497 into alanines (A); 2KR, point mutation of lysines (K) 494/497 into arginines (R); EGFP, enhanced green fluorescence protein; IF, immunofluorescence.

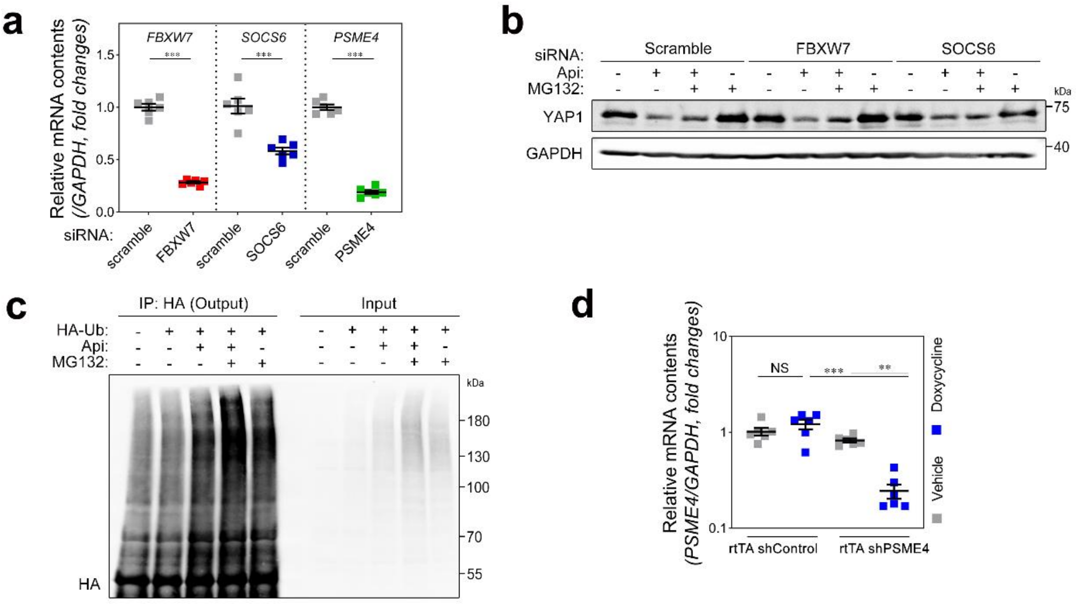

Supplementary Figure S5. Atypical degradation of YAP1 through PSME4. (a) Knock-down efficiency was confirmed by quantitative real-time PCR. (b) Apicidin was still effective for degradation of YAP1 even in the absence of FBXW7 or SOCS6. (c) Polyubiquitination assay. HA-Ubiquitin was precipitated with anti-HA antibody and visualized with HA antibody. Output signal represented (poly)ubiquitinylated proteins. Apicidin itself induced (poly)ubiquitinylation of various proteins, which was further augmented by MG132, a proteasome inhibitor. Note that input results also showed (poly)ubiquitinylation. (d) Doxycycline treatment knocked down endogenous PSME4. Data are denoted as mean ± standard error. Nonparametric Mann-Whitney U test (a) or two-way ANOVA and Tukey's HSD post hoc test was applied for statistical analyses (d). Asterisks: ** $p<0.01$, *** $p<0.001$, NS, not significant. Abbreviation: siRNA, small interfering RNA.

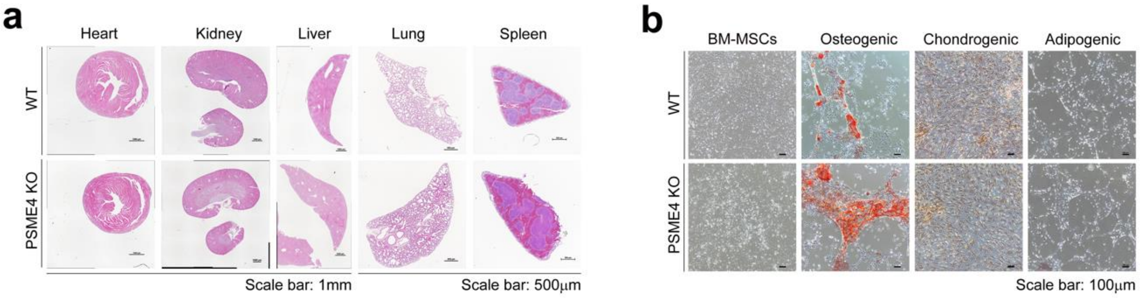

Supplementary Figure S6. PSME4 null mice. (a) Morphology of organs. The heart of PSME4-null mice is smaller than that of wild-type littermates. The other organs showed no differences. (b) Biased differentiation of PSME4 deletion. Primary cultures of bone marrow-derived mesenchymal stem cells of wild-type or PSME4 knockout littermate were prepared and differentiation properties were tested. Mesenchymal stem cells from PSME4 knockout greatly differentiated into osteogenic lineages. Otherwise, there were no differences. Abbreviation: BM-MSCs, bone marrow-derived mesenchymal stem cells; KO, knockout.

Supplementary Table S1. Anatomical parameters in each mouse groups.

Parameters					*p* value of main effect		
	1. Male/WT	2. Male/KO	3. Female/WT	4. Female/KO	Sex	Genotype	Interaction
Body Weight (g)	23.69 ± 0.47	24.65 ± 0.40	18.54 ± 0.41	18.25 ± 0.36	<0.001	0.452	0.165
Heart (mg)	115.78 ± 3.16	112.88 ± 2.08	91.65 ± 1.63	84.59 ± 1.75	<0.001	0.039	0.384
Liver (mg)	1243.4 ± 49.94	1254.30 ± 30.30	919.43 ± 28.78	885.98 ± 30.10	<0.001	0.759	0.547
Lung (mg)	142.89 ± 3.12	147.52 ± 2.44	127.50 ± 2.90	123.32 ± 2.67	<0.001	0.940	0.137
Kidney (mg)	296.55 ± 9.7	296.10 ± 4.98	224.38 ± 5.33	212.09 ± 4.20	<0.001	0.313	0.349
Spleen (mg)	80.40 ± 3.75	83.57 ± 2.13	75.30 ± 4.29	69.37 ± 2.66	0.011	0.902	0.080
HW/BW (mg/g)	4.88 ± 0.06	4.59 ± 0.06	4.96 ± 0.08	4.64 ± 0.06	0.353	<0.001	0.887
LiW/BW (mg/g)	52.54 ± 1.95	50.83 ± 0.74	49.71 ± 1.43	48.48 ± 1.08	0.039	0.235	0.845
LuW/BW (mg/g)	6.04 ± 0.10	6.00 ± 0.08	6.89 ± 0.14	6.77 ± 0.10	<0.001	0.427	0.686
KW/BW (mg/g)	12.49 ± 0.21	12.03 ± 0.14	12.13 ± 0.26	11.65 ± 0.18	0.061	0.017	0.947
SW/BW (mg/g)	3.39 ± 0.13	3.48 ± 0.07	4.04 ± 0.18	3.79 ± 0.11	<0.001	0.506	0.172

Data were presented in mean ± standard deviations. Absolute organ weight or normalized-organ weight dividing by individual body weight was presented. Two-way ANOVA. No further stratification was carried out because no significant interaction was determined. Abbreviations: BW, body weight; LiW, liver weight; LuW, lung weight; KO, knock-out; KW, kidney weight; SW, spleen weight; WT, wild type.
